# Nipah virus Malaysia and Bangladesh strain-induced pathogenesis in mice lacking type I interferon receptor signaling

**DOI:** 10.1371/journal.pntd.0013894

**Published:** 2026-08-20

**Authors:** Lucia Amurri, Olivier Reynard, Ilona Ronco, Daniel Déri, Bernadett Pályi, Julia Spanier, Jennifer Skerra, Olivia Terceve, Thibaut Larcher, Ulrich Kalinke, Zoltán Kis, Branka Horvat, Mathieu Iampietro

**Affiliations:** 1 CIRI, Centre International de Recherche en Infectiologie, INSERM U1111, CNRS, UMR5308, Univ Lyon, Université Claude Bernard Lyon 1, École Normale Supérieure de Lyon, Lyon, France; 2 National Biosafety Laboratory, National Center for Public Health and Pharmacy, Budapest, Hungary; 3 Semmelweis University, Institute of Medical Microbiology, Budapest, Hungary; 4 Institute for Experimental Infection Research, TWINCORE, Centre for Experimental and Clinical Infection Research, a Joint Venture between the Helmholtz-Centre for Infection Research and the Hannover Medical School, Hannover, Germany; 5 UMR703, PAnTher, APEX, Oniris VetAgroBio, La Chantrerie, Nantes, France; 6 Cluster of Excellence - Resolving Infection Susceptibility (RESIST, Hannover Medical School, EXC 2155), Hannover, Germany; 7 European Research Infrastructure on Highly Pathogenic Agents (ERINHA-AISBL), Brussels, Belgium; Seoul National University College of Medicine, KOREA, REPUBLIC OF

## Abstract

Nipah virus (NiV) is a zoonotic highly pathogenic *Paramyxovirus* inducing lethal outbreaks of encephalitis and Acute Respiratory Distress Syndrome (ARDS) with an average case-fatality rate of 75%. Two viral strains, NiV-Malaysia (NiV-Mal) and NiV-Bangladesh (NiV-Ban), associated to distinct route of transmission, symptoms and lethality have been described. Due to the permanent threat of these emerging infections and the lack of approved therapeutics, it is crucial to improve our understanding regarding NiV-associated pathogenesis. Mice represent a small and accessible animal model, provided with numerous biological tools for the functional assessment of different genes related to antiviral response. Here, we explore the susceptibility of mice deficient for type I interferon receptor (IFNAR KO) to inoculation with either NiV-Mal or NiV-Ban through intraperitoneal or intranasal routes. Our results complement observations showing that IFNAR KO mice are susceptible to NiV-Ban infection via intraperitoneal route, although to a lesser extent than NiV-Mal, and develop encephalitis and a pulmonary syndrome with viral dissemination to various organs. Additionally, intranasal administration of both viral strains exhibited a subclinical infection with viral replication in the brain and the lungs along to the production of neutralizing antibodies in some animals. These results suggest that IFNAR KO mice may represent a reliable model permitting comparative studies of the immunopathogenesis induced by both NiV-Mal and NiV-Ban infections.

## Author summary

Availability of small animal models represents a major issue to characterize virus-associated pathogenesis to further implement therapeutic strategies. Previous studies performed with NiV-Mal and NiV-Ban strains in wild-type (WT) mice did not indicate signs of NiV disease. However, infection of IFNAR KO mice with NiV-Mal resulted in fatal outcome, thus providing useful information on viral immunopathogenesis. Surprisingly, while NiV-Ban being the viral strain re-emerging the most frequently, studies involving NiV-Ban in IFNAR KO mice were lacking. Here, we compared pathogenesis between NiV-Mal- and NiV-Ban-infection following IP and IN inoculations in IFNAR KO animals. Indeed, our results pointed that contrary to fatal IP challenge, IN inoculation was associated to a subclinical infection with both NiV strains. Moreover, we noticed that NiV-Mal tends to be more pathogenic than NiV-Ban following IP inoculation. Finally, distinct histopathological manifestations and tropism were observed with each viral strain and specific route of infection. Overall, our study supposes that IFNAR KO mice may represent a useful animal model to study strain-specific NiV pathophysiology.

## Introduction

Nipah virus (NiV) is an emerging zoonotic *Paramyxovirus* causing highly lethal outbreaks in South-East Asia, Bangladesh and India [1]. NiV is naturally hosted by *Pteropus* fruit bats, which are widespread in tropical and subtropical areas of Asia and Indian/Pacific Ocean islands and Australia [2,3]. Due to its high pathogenicity, its human-to-human transmission and the lack of approved treatments or vaccines, World Health Organization has included NiV in its Blueprint list of epidemic threats requiring urgent research and development actions [4]. Moreover, climate change, deforestation and intensive farming are progressively disrupting the habitats of reservoir animals and increasing animal-human contacts, leading to an increased risk for epidemic or pandemic [5–9].

Two NiV strains have been described, NiV Malaysia (NiV-Mal) and NiV Bangladesh (NiV-Ban), that share 91.8% of nucleotide sequence homology but show remarkable differences in epidemiology and human pathology [8,10]. The first NiV outbreak was registered in Malaysia in 1998–1999, where the virus was first transmitted from asymptomatic fruit bats to pigs as intermediate hosts before infecting humans [11]. This led to 265 cases of acute encephalitis and 105 deaths (case fatality rate, CFR = 40%), mostly among pig farmers who were exposed to infected animals.

A second NiV strain emerged in Bangladesh in 2001 through a direct spillover from bats to humans following the consumption of contaminated date palm sap [12]. Indeed, in this geographical area sap is harvested from palm trees using reservoirs containing juice that bats can approach and contaminate with saliva and/or urine [13]. Moreover, in addition to animal-to-human transmission, respiratory human-to-human transmission has been reported [14]. While NiV-Mal is commonly associated with lethal encephalitis, NiV-Ban patients mainly display severe respiratory syndrome in addition to neurological symptoms. Since 2001, NiV-Ban is re-emerging frequently in Bangladesh and India, with an average CFR of approximately 75%, reaching up to 100% in some outbreaks and representing a constant threat related to new episodes or potential pandemic spread [15–17].

NiV can infect a wide range of mammals by interacting with the highly-conserved and ubiquitously expressed Ephrin B2 and B3 receptors [18–20]. However, while humans, pigs and different animal models such as non-human primates (NHP), hamsters and ferrets, develop clinical signs of disease associated with high lethality following NiV infection, mice clear the virus despite being permissive to infection [21–23]. To efficiently and better characterize viral pathogenesis, convenient animal models are needed. Indeed, while several studies using hamsters as a model have allowed the establishment a pre-clinical approach including both NiV strains and both intraperitoneal (IP) and intranasal (IN) routes of inoculation [24], the development of an appropriate mouse model would represent an important progress to further decipher NiV pathogenesis *in vivo*.

In previous studies addressing NiV-Mal infection in both wild type (WT) and Interferon α/β receptor knock out (IFNAR KO) mice, all WT animals survive both IP and IN challenges while 100% and 60% of IFNAR KO mice succumb to NiV-Mal challenge, respectively [23,25–27]. In addition, viral dissemination in the brain, the lungs, the spleen and the liver, along brain-associated lesions, leading to neurological signs of disease, were observed in IP-inoculated IFNAR KO mice [25]. Afterwards, other works have been conducted on NiV infection in WT mice [28,29], notably Dups *et al.* compared NiV-Mal and NiV-Ban infections in intranasally inoculated WT mice [28]. All animals survived without developing any sign of disease, and both viral RNA and viral antigens were detected in the lungs of infected animals only, suggesting that WT mice were able to control viral dissemination, with no significant differences between viral strains. Moreover, no neutralizing antibodies (nAb) response was detected. These observations were recently confirmed by Edwards *et al.*, who analyzed NiV-Ban IN inoculation in WT mice with all animals surviving without displaying either clinical signs of disease or histological lesions, and viral detection in the lungs only and a mild production of nAb in 3 out of 5 mice [29].

Contrary to NiV-Mal, NiV-Ban infection has not been extensively examined in IFNAR KO mice [30] and further investigations have been required to better understand differential pathogenesis between those two NiV strains. Here, we conducted exploratory studies aimed at comparing pathogenesis in both WT and IFNAR KO mice infected with NiV-Mal or NiV-Ban through IP and IN inoculations.

First, we evaluated the replication and viral expansion in primary murine embryonic fibroblasts (MEFs) derived from WT and IFNAR KO mice to both NiV strains *in vitro*, and observed a higher efficiency for NiV-Mal to replicate compared to NiV-Ban in both MEFs genotypes. Then, we analyzed the susceptibility of both mouse genotypes to NiV-Mal or NiV-Ban following IP or IN inoculation *in vivo*. We endorsed concomitant observations that NiV-Mal was more virulent than NiV-Ban in IP-inoculated IFNAR KO mice [30]. Moreover, we confirmed that neither NiV-Mal nor NiV-Ban lead to fatal outcome following IN inoculation in IFNAR KO mice. Furthermore, viral genome quantification and histopathological analysis were performed on harvested organs to determine the magnitude of viral spread and tropism. While IN inoculation was associated to a subclinical infection in all experimental groups, some IFNAR KO-IP-inoculated animals exerted higher viral replication and tissular pathology compared to WT mice. Interestingly, different cellular tropism and syncytia distribution could be observed in the lungs of IFNAR KO animals depending on the viral strain that was inoculated.

Globally, our work presents preliminary inputs concerning comparative studies related to NiV pathogenesis and paves the way for further use of IFNAR KO mice as a small animal model for fundamental and pathophysiology studies of both NiV-Mal and NiV-Ban strains, allowing thus to take advantage of numerous biological tools available in murine studies.

## Materials and methods

### Ethical statement

Animal experiments were performed in the biosafety level 4 (BSL-4) facility of the National Biosafety Laboratory in Budapest according to the guidelines of the European Communities Council Directive (86/609 EEC) and were approved by the Hungarian National Authority (Scientific Ethics Council for Animal Experiments, PE/EA/1456–7/2020).

Animals in transit at Plateau de Biologie experimentale de la souris (PBES) in Lyon were manipulated in accordance to good experimental practice and approved by the regional ethics committee CECCAPP (Comité d’Evaluation Commun au Centre Léon Bérard, à l’Animalerie de transit de l’ENS, au PBES et au laboratoire P4) and authorized by the French Ministry of Higher Education and Research (no. 00962.01).

### Mice

Wild type (WT) C57BL/6J mice (Charles River) and B6.129S2-Ifnar1tm1(Neo)Agt (IFNAR-KO) mice (Jackson Laboratory) [31] were bred under specific pathogen-free conditions in the central mouse facility of the Helmholtz Centre for Infection Research (Brunswick), at TWINCORE (Centre for Experimental and Clinical Infection Research, Hanover, Germany) and at Plateau de Biologie experimentale de la souris (PBES) in Lyon. Mouse experimental work was carried out using 3-week-old to 6-week-old mice with equal male-female distribution. Mice were kept under BSL-4 conditions in individually ventilated cages (IsoRat ISO48NFEEU, Techniplast, Italy), housed on autoclaved corn bedding (Rehofix, J. Rettenmaier & Söhne GmbH + CO KG, Rosenberg, Germany), fed autoclaved food (VRFI (P), Special Diet Services) ad libitum, given osmosis-treated water provided in bottles ad libitum, and the cages were equipped for proper environmental enrichment.

Different groups have been established and are presented in S1 Fig Indeed, while all animals IP- or IN-inoculated with NiV-Mal were monitored for 32 days or euthanized if humane endpoint was reached (5 or 6 animals per group), the inoculations involving NiV-Ban were divided in two categories, one involving the same 32-days monitoring (5 animals per group) and another one involving a kinetic with programmed euthanasia at day 0 + 4h, day 1, day 2, day 4, day 7 and day 10 (2 or 3 animals per time point). Moreover, to match and compare the kinetic with studies performed for NiV-Mal [27], the samples harvested from NiV-Ban-challenged animals were pooled into early (from day 0 + 4h to day 1), mid (from day 2 to day 7) and late (from day 8 day 32) phases of infection (S1 Fig).

Clinical scores were established following a specific table implemented to evaluate henipavirus infections in mice [32] already used in previous studies [23,26,27].

### Cell lines

WT or IFNAR KO primary murine embryonic fibroblasts (MEFs) were isolated from murine embryos obtained from female pregnant mice 13 days after conception [26,33] and cultured in Dulbecco’s modified Eagle’s medium (DMEM) GlutaMAX supplemented with 10% heat-inactivated fetal bovine serum (FBS), 0.2% 2-mercaptoethanol, 1% HEPES, 1% nonessential amino acids, 1% sodium pyruvate, and 2% penicillin-streptomycin mix at 37°C with 5% CO2. MEFs were tested negative for mycoplasma (MycoAlert, Lonza).

WT and IFNAR KO MEFs were plated in 12-well plates (1.5x10^5^ cells/well) or in 24-well plates (5x10^4^ cells/well) for immunofluorescence or RT-qPCR analysis, respectively. MEFs were then infected in the Jean Mérieux BSL-4 facility in Lyon with NiV-Mal or NiV-Ban at a multiplicity of infection (MOI) of 0.3 plaque-forming units (PFU)/cell (according to the viral titer previously calculated on Vero E6 cells) for 1h at 37°C in DMEM 0% FBS. Then, virus-containing medium was removed and substituted with fresh complete medium before being incubated for 24h or 48h at 37°C.

### Viruses

NiV-Mal (isolate UMMC1 GenBank-AY029767, kindly provided the University of Malaya, Kuala Lumpur, Malaysia) and NiV-Ban (isolate SPB200401066 GenBank-AY988601, kindly provided by the Center for Disease Control and Prevention, Atlanta USA) were prepared by infecting Vero E6 cells cultured in DMEM, in the INSERM Jean Mérieux BSL-4 in Lyon, France and in the National Biosafety Laboratory in Budapest, Hungary. Both viral stocks were titrated by plaque assay on VeroE6 cells. Each viral strain used for inoculations consists in a “passage P3” from stocks already validated and used in previous works [26,27,34,35]. Both viral stocks were tested negative for mycoplasma (MycoAlert, Lonza).

### Intraperitoneal and intranasal inoculation of mice

All NiV-Mal and NiV-Ban inoculations were performed under BSL-4 conditions. Viral inoculums were prepared at 10^5^ PFU for intranasal (IN) inoculation and 10^6^ PFU for intraperitoneal (IP) inoculation in DMEM media and tittered before and after infections. Mice were inoculated intranasally with 10^5^ PFU of NiV in 50 µL DMEM and intraperitoneally with 10^6^ PFU of NiV in 200 µL DMEM under inhalational anesthesia with isoflurane (isoflurane: ISOFLUTEK 1000 mg/g, Laboratorios Karizoo S.A.; anesthesia station: MiniHUB-V3, TEM SEGA, France).

Following inoculation, animals were weighed and monitored on a daily basis as part of a scoring system to follow the signs of disease and determine endpoint euthanasia. After NiV-Mal inoculation, animals were monitored for 32 days and organs were collected at the end of the experiment or at the day of death. For NiV-Ban inoculation, animals were monitored during 1-month post-infection, and in addition programmed euthanasia was performed for 4 hours, 1 day, 2 days, 4 days, 7 days or 10 days post-infection (d0 + 4h, d1, d2, d4, d7, d10), and at the end of the protocol in case of mice which survived infection. For graphical representations of qPCR data and statistical analyses, murine samples were pooled and grouped into early (from d0 + 4h to d1), mid (from d2 to d7) and late (after d7 until d32) phase of infection.

Euthanasia was performed when a humane endpoint was reached or at the end of the study, 32 days after infection. Necropsies were performed and the organs fixed using 4% formaldehyde (Merck, USA) during two series of seven days in BSL4 conditions and then processed for histopathological analysis.

### Sample collection and preparation

Euthanasia with CO_2_ was conducted under inhalational anesthesia with isoflurane, followed by blood collection via cardiac puncture and autopsy. During autopsy, brains, lungs, spleens and livers were collected for further nucleic acid isolation and histological analysis. Organs were divided for molecular testing and were collected and frozen immediately at -80°C without any medium until further preparation. Parts of organs for immunohistochemistry were collected into fixative 10 V/V% formaldehyde-PBS solution. Then, 25–30 mg of the thawed organs were taken into MagNA Lyser Green Beads tubes (Roche Diagnostics GmbH, Mannheim, Germany) containing 600 µL of 1:100 mixture of β-mercaptoethanol and Buffer RLT (QIAGEN GmbH, Hilden, Germany). After homogenization using MagNA Lyser instrument (Roche Diagnostics GmbH), samples were centrifuged for 5 minutes at 12000 rpm. Supernatants were transferred into new tubes and after 10 min of incubation at room-temperature, an equal volume of 70 V/V% ethanol was added into each tube.

### Seroneutralization

Seroneutralization assay was performed on mice euthanized after day 7. Serum samples were stored at -80^o^C without previous thawing and were analyzed without heat inactivation. Serum dilution and assay procedures were done according to manufacturer’s instructions under BSL-4 conditions. Sera were diluted in 7-point serial two-fold dilutions in triplicate in serum-free DMEM in sterile 96-well microtiter plates. Positive and negative control sera were also applied, the latter with and without serum as well. Equal volume of 60 ± 20 TCID50 NiV-Mal or NiV-Ban was added into each well and incubated for 1 h at 37^o^C. Then, samples were transferred on a Vero E6 cells monolayer (approximately 1,8x10^5^ cells/mL) maintained in serum-free DMEM in 96-well plates (TPP, Switzerland). The neutralizing antibody titer of each sample was determined by the lack of cytopathic effect (CPE) after a 5 days incubation at 37^o^C. For triplicates the geometric mean of end dilutions was calculated and reported as a neutralizing antibody titer (nAb).

### RNA extraction and RT-qPCR

Regarding murine samples, total nucleic acid extraction was performed using QIAsymphony SP instrument with QIAsymphony DSP Virus/Pathogen Mini Kit and Complex200_OBL_V4_DSP protocol (QIAGEN GmbH, Hilden, Germany). Quality control of samples qPCR was performed targeting the Peptidylprolyl Isomerase A (Ppia) housekeeping gene of the mouse as previously described [36]. The reaction was performed on LightCycler 480 Platform II using LightCycler Multiplex RNA Virus Master kit (Roche Lifesciences), and data were analyzed with LightCycler 480 SW 1.5.1 software using Abs Quant/2nd Derivative Max method.

Concerning *in vitro* MEF experiments, cells were collected at indicated time points and RNA was extracted using NucleoSpin RNA Kit (Macherey Nagel) or QIAamp Viral RNA mini-Kit (Qiagen) on cells and supernatants, respectively, according to manufacturer's instructions.

Extracted RNA was amplified by real-time (RT) quantitative (q) PCR using Luna Universal One-Step RT-qPCR Kit (NEB) on a StepOnePlus Real-Time PCR System. Pfaffl Model [37] and Bustin MIQE checklist [38] were used for all validations and calculations. To validate our primers, a PCR on a positive control has been performed before dosing 10^-1^ to 10^-12^ 10-fold dilutions of our PCR products using the Denovix DS-11-FX spectrophotometer to evaluate the quantity of DNA in each dilution. Then, the serial dilutions were run in qPCR to validate efficacy, specificity and sensitivity of each couple of primers. Moreover, the exact number of copies in each qPCR reaction was obtained by calculating the “N0 Samples = N0 Standard * Efficacy-ΔCt” before being converted using molecular weight of each targeted gene and Avogadro number. Results obtained were converted to “copies/μg of RNA” for cell lysate samples. Finally, normalization was performed by dividing obtained numbers of RNA copies of the target genes with the deviation of the mouse glyceraldehyde 3-phosphate dehydrogenase (mGAPDH) used as housekeeping gene. Specific set of primers were designed and validated for the detection of mGAPDH (forward: GCATGGCCTTCCGTGTCC; reverse: TGTCATCATACTTGGCAGGTTTCT) and viral NiV-N (forward: GGCAGGATTCTTCGCAACCATC, reverse: GGCTCTTGGGCCAATTTCTCTG).

### Immunofluorescence

MEFs cultured in 12-well plates were washed with PBS 1X and fixed with PFA 4% for 20 min at 4°C. After 2 washes with PBS 1X, cells were permeabilized with TritonX100 0.1%-PBS for 10 min and blocked with of bovine serum albumin (BSA) 2.5%-PBS for 10 min. After 2 washes with PBS-2% FCS-0.1% Tween, cells were incubated 1h at room temperature (RT) with an in-house produced rabbit anti-NiV nucleoprotein (NiV-N) I Ab 1/300 in PBS-2% FCS-0.1% Tween. Plates were then washed 2 times with PBS-2% FCS-0.1% Tween, incubated 1 h at room temperature with a mix of fluorophore-conjugated secondary antibody (anti-mouse AF488, Invitrogen) and DAPI (Merck) 1:1000. Finally, slides were washed 2 times with PBS-2% FCS-0.1% Tween, once with PBS 1X, covered in PBS 1X and stored at 4°C. Fluorescence was evaluated using a Zeiss Axio OBSERVER Z.1 microscope, and photographs were treated using ImageJ software version Java 1.8.0_112.

### Histology and immunohistochemistry

Regarding histopathological evaluation, formalin-fixed specimens were embedded in paraffin wax, 4 μm tissue sections were processed and stained with hematoxylin-eosin-saffron (HES). Immunohistochemistry was performed using the Ventana Discovery Ultra Instrument (Ventana Medical Systems, Roche Diagnostics). The sections were dewaxed and rehydrated. Slides were pre-treated with CC1 (pH 8, Roche Diagnostics) at 98 °C for 40 min. They were then incubated for 30 min at room temperature with blocking buffer (Diagomics), followed by a 1-hour incubation at 37 °C with the primary antibody, rabbit polyclonal anti-NiV-N (1:250). After rinsing, slides were incubated for 30 min at room temperature with a biotinylated goat anti-rabbit IgG antibody (1:200, Dako). Immunoreactive complexes were visualized using streptavidin-conjugated horseradish peroxidase (1:200, Dako) and DAB chromogen (Fisher Scientific). Sections were counterstained with Hematoxylin II and examined using a Nikon Eclipse Ni microscope equipped with a Nikon DS-Ri color camera. Histopathological analysis was performed by a board-certified veterinary pathologist.

## Results

### NiV-Mal replicates at higher levels compared to NiV-Ban in IFNAR KO primary murine cells

**Fig 1:**
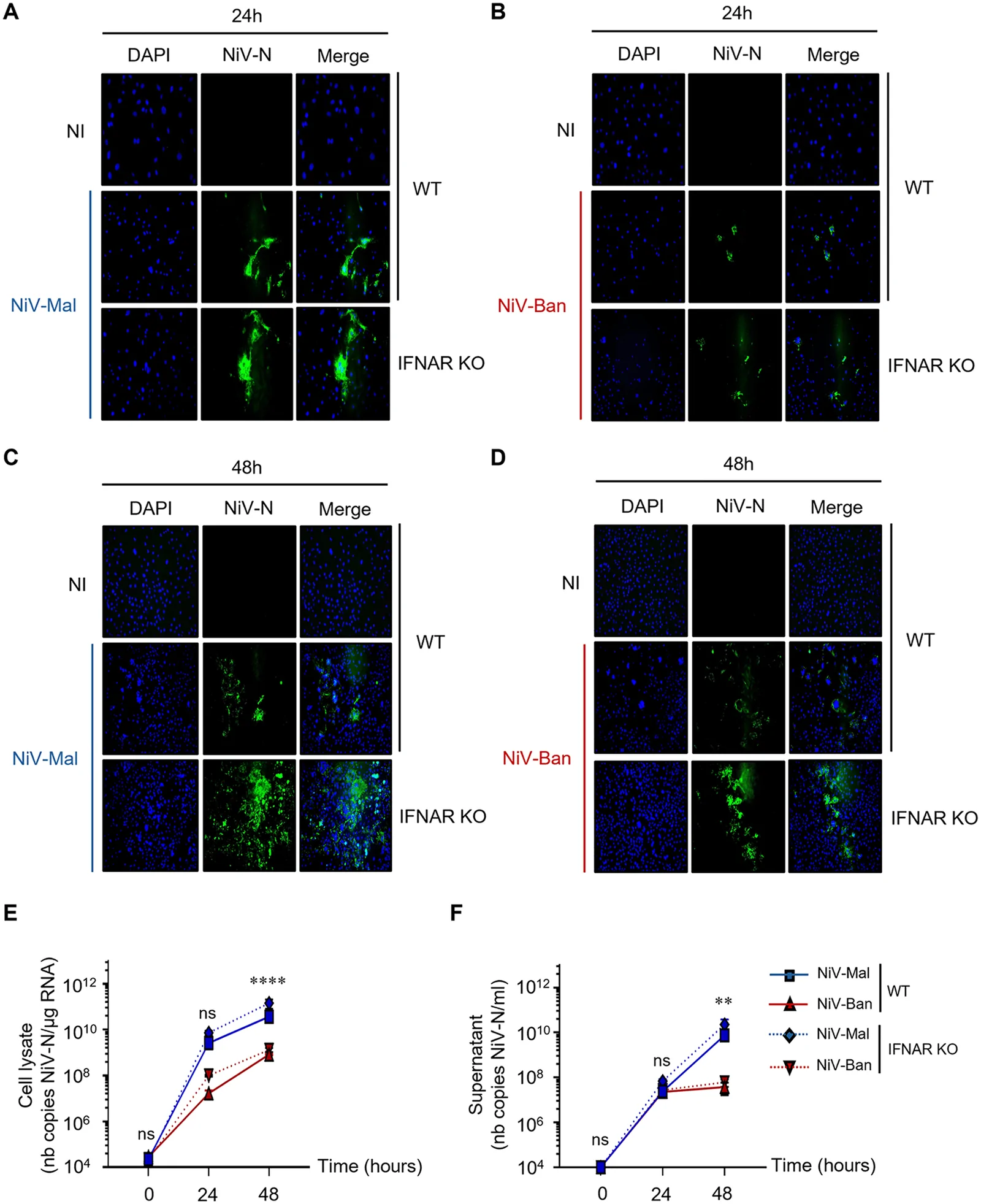
NiV-Mal replication is more efficient than NiV-Ban in primary murine cells. Murine embryonic fibroblasts (MEFs) derived from WT or IFNAR KO mice were non-infected (NI) or infected with NiV-Mal or NiV-Ban at a MOI of 0.3 and then cultured for 0h, 24h or 48h (n = 3). **(A-D)** Cells were fixed and stained with DAPI and an anti-NiV nucleoprotein (NiV-N) antibody before being evaluated by fluorescence microscopy. **(E-F)** NiV-N mRNA levels were assessed from cell lysates and supernatants by real time RT-qPCR. Data are represented as mean + standard deviation (SD) of three independent replicates. The difference between NiV-Mal and NiV-Ban in IFNAR KO mice was analyzed using two-way analysis of variance, followed by a Tukey multiple comparison test: ns (not significant); ****p < 0.0001.

The replication and cytopathic effects induced by NiV-Mal and NiV-Ban were initially analyzed *in vitro* in MEFs obtained from WT and IFNAR KO mice sharing the same genetical background employed in further *in vivo* experiments. Both MEFs genotypes were infected with either NiV strains (multiplicity of infection (MOI) of 0.3) and both cell lysates and supernatants were collected immediately after viral inoculation (0h) and then at 24h and 48h post-infection (Fig 1). NiV-N protein and mRNA levels were analyzed through immunofluorescence (Fig 1A-1D) and RT-qPCR (Fig 1E and 1F), respectively. Both cell genotypes were permissive to both NiV strains, although NiV-Ban demonstrated a reduced replication compared to NiV-Mal (Fig 1A-1F). Also, both viral strains replicated at higher levels in IFNAR KO cells compared to WT MEFs (Fig 1A-1D). Moreover, multiple NiV-Mal-induced syncytia appeared 24h post-infection and progressively expanded until 48h post-infection (Fig 1A and 1C). Conversely, syncytial multi-nucleated cells were observed only at 48h following NiV-Ban infection (Fig 1B and 1D) associated with a lower replication rate compared to NiV-Mal infected-cells (Fig 1E and 1F). Globally, the replication of NiV-Mal was more efficient than NiV-Ban in primary MEFs, resulting in higher levels of viral genomes both produced in the cells and released in the supernatant (Fig 1A-1F).

### NiV-Mal challenge leads to more severe outcome compared to NiV-Ban in IFNAR KO mice

**Fig 2:**
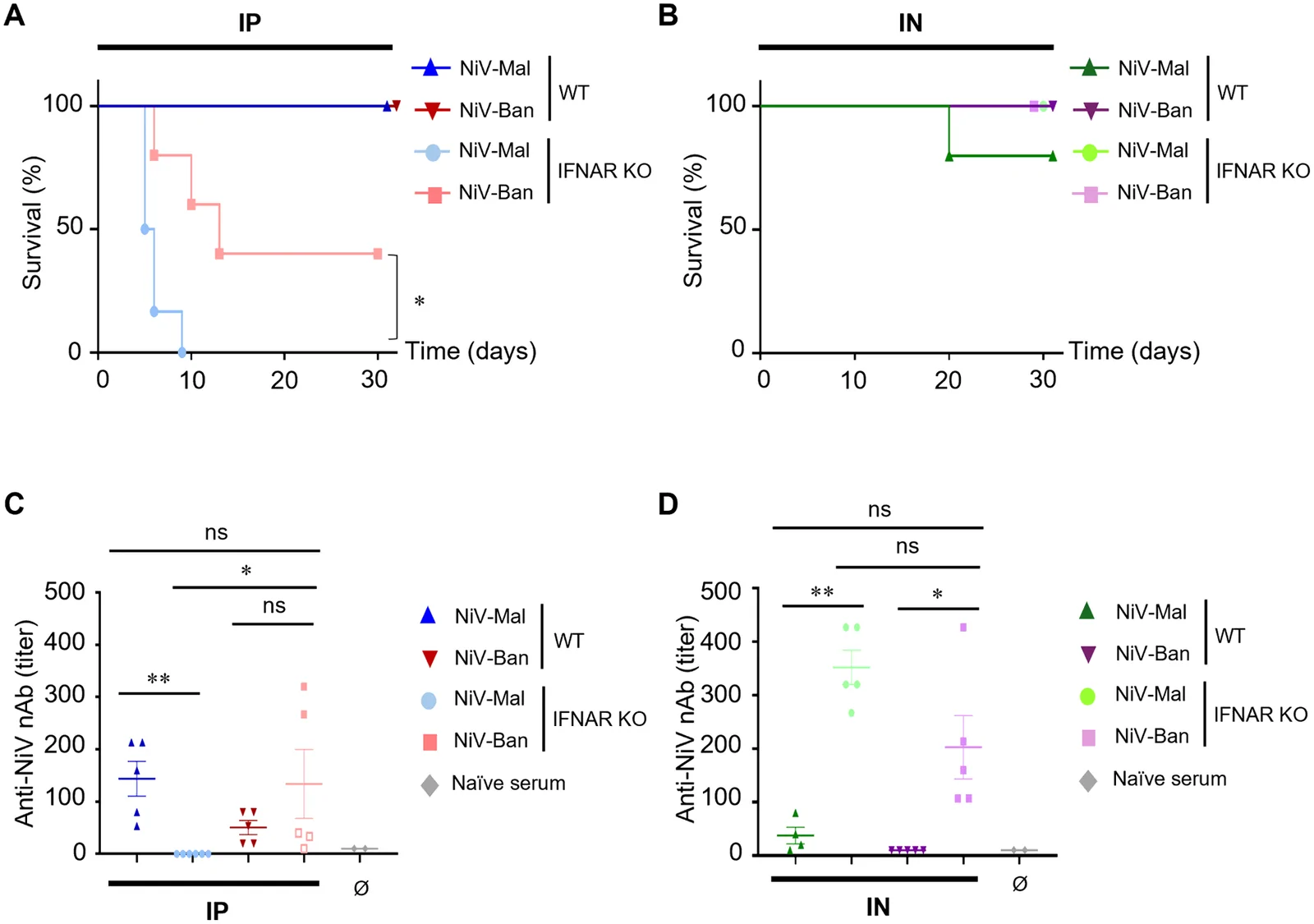
NiV-Mal and NiV-Ban induce different disease severity in mice. WT and IFNAR KO mice were infected through intraperitoneal route (IP) or intranasal route (IN) with 10^6^ or 10^5^ plaque forming units (PFU), respectively, of NiV-Mal or NiV-Ban. All groups were constituted of 5 animals except for the NiV-Mal IFNAR KO group, that contained 6 mice. **(A-B)** Survival of infected mice was followed up to 32 days. Survival was analyzed using Gehan-Breslow-Wilcoxon test. *p < 0.05. **(C-D)** Virus-specific nAb titer was evaluated by seroneutralization in sera collected at the day of euthanasia (empty dots) or at the end of the experiment (full dots). Serum could not be collected in the case of NiV-Mal-IN-inoculated mouse that was found dead at d20. Horizontal lines correspond to the average nAb titer of each group. ND = non detected. Data are represented as mean ± standard error mean (SEM). Samples were analyzed using Kruskal Wallis and Dunn’s multiple comparison test, *p < 0.05, **p < 0.01.

We next compared NiV-Mal and NiV-Ban infection in murine models *in vivo.* Indeed, we challenged WT and IFNAR KO mice through IP and IN route with 10^6^ and 10^5^ PFU of virus, respectively and monitored the animals for 32 days (Figs 2 and S2).

While all WT mice survived IP inoculations, all NiV-Mal IP-infected IFNAR KO animals succumbed at day 6 ± 1.4 days, while NiV-Ban IP infection provoked the death of 60% of mice at day 9.6 ± 2.9 (Fig 2A). Both NiV-Mal- and NiV-Ban-inoculated mice that succumbed to the infection showed similar signs of disease, including loss of weight, ruffled hair, facial pain and paralysis (S2A-S2D Fig). Moreover, 3 NiV-Mal-IP-inoculated IFNAR KO mice that died at day 5 or day 6 displayed breathing difficulties (S2C Fig). In contrast, all IN-inoculated IFNAR KO mice survived until the end of the experiment (Fig 2B). Intriguingly, one IN-inoculated WT mouse succumbed to NiV-Mal infection and was found dead at d20 (Fig 2B), despite displaying any sign of disease throughout the experiment (S2D Fig). Following IP inoculation with either viral strain, all WT mice produced specific anti-NiV neutralizing antibodies (nAb) (Fig 2C). Also, NiV-Mal-IP-inoculated IFNAR KO animals did not produce nAb, consistent with the fact that they all died before day 10, thus before being able to develop a humoral response (Fig 2C). Concerning NiV-Ban-IP-inoculated mice, both animals surviving viral challenge produced nAb, thus confirming their initial infection (Fig 2C). Finally, following IN inoculation with either viral strain, IFNAR KO mice produced significantly more nAb than WT mice (Fig 2D). Overall, this exploratory analysis highlights a more aggressive infection by NiV-Mal compared to NiV-Ban in IFNAR KO mice.

### NiV-Mal tends to disseminate more than NiV-Ban after intraperitoneal inoculation in IFNAR KO mice

**Fig 3:**
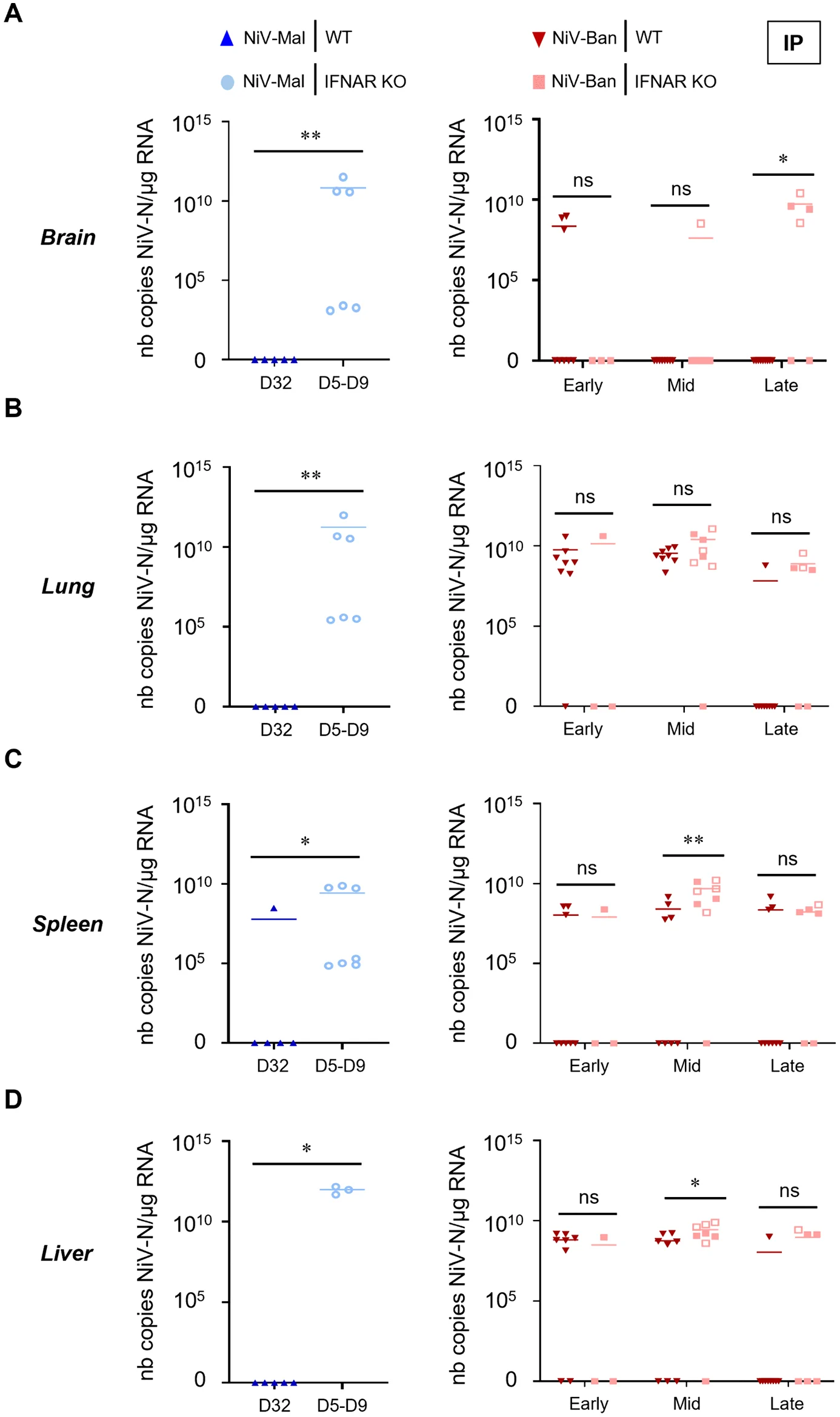
Intraperitoneal inoculation of NiV features viral dissemination in IFNAR KO mice. **(A-D)** WT (up blue triangle and down red triangle) and IFNAR KO (light blue circle ang light red square) mice were inoculated intraperitoneally with 10^6^ PFU of NiV-Mal (A-D, left graphs) or NiV-Ban (A-D, right graphs). Following NiV-Mal inoculation, NiV nucleoprotein (NiV-N) mRNA levels in murine brains (A, left), lungs (B, left), spleens (C, left) and livers (D, left) were harvested either at d32 from surviving WT mice (full dots) or at the day of death for IFNAR KO mice (empty dots) and were assessed by RT-qPCR. Following NiV-Ban inoculation, NiV-N mRNA levels in murine brains (A, right), lungs (B, right), spleens (C, right) and livers (D, right) were harvested at the day of death (empty dots) or programmed euthanasia (full dots) and were assessed by RT-qPCR. For graphical representation and statistical analyses, mice samples were pooled into early (from d0 + 4h to d1), mid (from d2 to d7) and late (from d8 to d32) phase of infection. Horizontal lines correspond to the average of each group. The statistical difference between IFNAR KO and WT mice groups was analyzed using Mann Whitney test: ns (not significant); *p < 0.05; **p < 0.01.

To explore and investigate viral propagation, the presence of NiV-N RNA was examined in the brain, lung, spleen and liver of non-perfused infected animals (Fig 3). As we have previously analyzed NiV-Mal infection in details [25], here only one group of NiV-Mal-infected animals was included and monitored during one month (Fig 3A-3D, left panels). As extensive studies of NiV-Ban in IFNAR KO mice were lacking, two groups of animals were IP-inoculated with the virus to characterize early and later events separately. For biological accuracy and consistency with our previous study [27], RT-qPCR data from the two groups of NiV-Ban-infected mice were pooled and represented as “early” (+4h - d1), “mid” (d2 - d7) and “late” (d8 - d32) periods (Fig 3A-3D, right panels).

Despite notable differences in disease severity, both strains of NiV were detected in the brain (Fig 3A), lung (Fig 3B), spleen (Fig 3C) and liver (Fig 3D) of IP-inoculated IFNAR KO mice during the course of infection. However, while high levels of NiV-N RNA were detected in each organ of all NiV-Mal-IP-inoculated IFNAR KO mice, only few NiV-Ban-IP-inoculated animals were positive throughout the whole challenge. In parallel, no viral RNA was detected in any tested organ of WT animals infected with NiV-Mal and analyzed at d32 following complete survival, thus characterizing a potential effective viral control (Fig 3A-3D, left panels) and confirming our previous observations where neither viral RNA nor viral antigen were detected at the late period [27]. Also, we observed that some NiV-Ban-IP-inoculated WT mice showed active viral replication throughout early, mid and late phases in the three scrutinized organs, with almost all animals clearing the infection by d32 (Fig 3A-3D, right panels). Intriguingly, three WT mice were positive for NiV-Ban in the brain in the early phase of infection while viral RNA was no longer detected in the brain at mid and late phases anymore (Fig 3A, right panel). Interestingly, among IFNAR KO brain samples evaluated, 6 out of 6 were virus-positive after NiV-Mal infection, while only 5 out of 17 were virus-positive following NiV-Ban infection (Fig 3A, left panel and 3A, right panel), thus confirming the greater capacity of NiV-Mal to reach the brain compared to NiV-Ban. Overall, these observations describe a possibly wider spread of NiV-Mal compared to NiV-Ban in IP-inoculated IFNAR KO mice.

### NiV-Mal and NiV-Ban dissemination remain limited following intranasal inoculation in IFNAR KO mice

**Fig 4:**
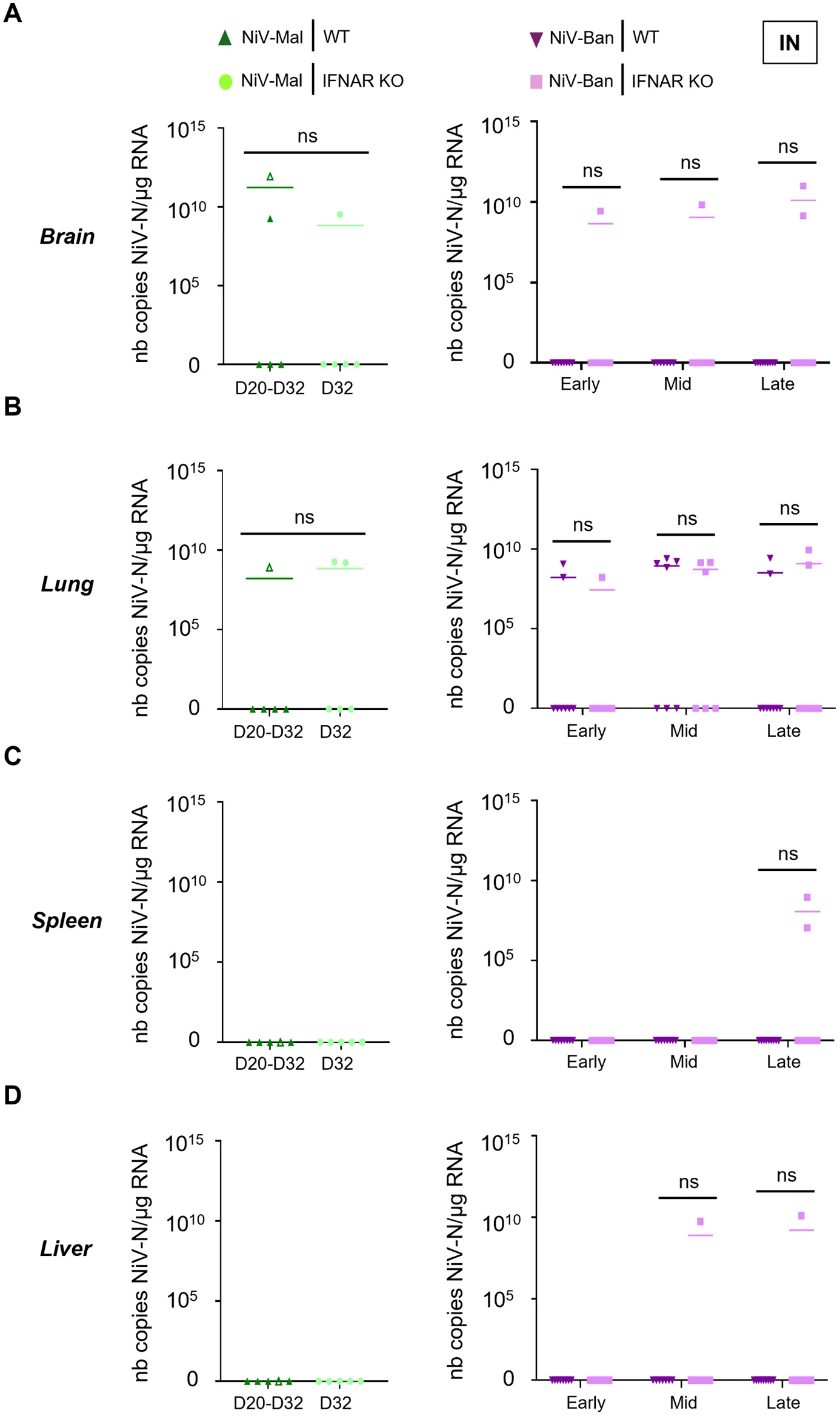
Intranasal NiV infection is controlled in IFNAR KO mice. **(A-D)** WT (up green triangle and down purple triangle) and IFNAR KO (light green circle ang light purple square) mice were inoculated intraperitoneally with 10^5^ PFU of NiV-Mal (A-D, left graphs) or NiV-Ban (A-D, right graphs). Following NiV-Mal inoculation, NiV nucleoprotein (NiV-N) mRNA levels in murine brains (A, left), lungs (B, left), spleens (C, left) and livers (D, left) were harvested either at the day of death (d20, empty dot) or at d32 from surviving WT mice (full dots) and IFNAR KO mice (full dots) before being assessed by RT-qPCR. Following NiV-Ban inoculation, NiV-N mRNA levels in murine brains (A, right), lungs (B, right), spleens (C, right) and livers (D, right) were harvested at day 32 (full dots) and were assessed by RT-qPCR. For graphical representation and statistical analyses, mice samples were pooled into early (from d0 + 4h to d1), mid (from d2 to d7) and late (from d8 to d32) phase of infection. Horizontal lines correspond to the average of each group. The statistical difference between IFNAR KO and WT mice groups was analyzed using Mann Whitney test: ns (not significant).

Following IN inoculation with either NiV strain, most animals were negative for NiV-N RNA, consistent with the fact that, despite one fatal outcome, all other animals survived without major clinical signs of disease (Fig 4). Interestingly, the WT mouse that succumbed to NiV-Mal IN inoculation at d20 (Fig 2B) was found positive for NiV-N RNA in both the brain and the lungs (Fig 4A and 4B, left panels). Then, contrary to IP-inoculated animals, no significant differences were detected between IN-inoculated WT and IFNAR KO mice (Fig 4A-4D, left and right panels). Moreover, the most affected organ was the lung, where viruses were detected in approximately 30% of animals independently of the viral strain (3/10 NiV-Mal-IN-inoculated mice and 14/45 NiV-Ban-IN-inoculated mice) (Fig 4B, left and right panels). Spleens and livers were all negative for NiV-Mal RNA (Fig 4C and 4D, left panels) and only two IFNAR KO mice were positive for NiV-Ban at mid/late time points, potentially consistent with the IN route of inoculation (Fig 4C and 4D, right panels). Additionally, the brains of two WT and one IFNAR KO animals IN-inoculated with NiV-Mal were positive for NiV-N by RT-qPCR (Fig 4A, left panel), while the four samples positive following NiV-Ban infection experiment were collected from IFNAR KO mice only (Fig 4A, right panel). Globally, our initial data tend to describe a more limited spread of either NiV strain following IN inoculation consistent with the higher survival rate of challenged IFNAR KO mice (Fig 2).

### Intranasal inoculation of both NiV-Mal and NiV-Ban induces milder immunopathogenesis compared to intraperitoneal inoculation in IFNAR KO mice

**Fig 5:**
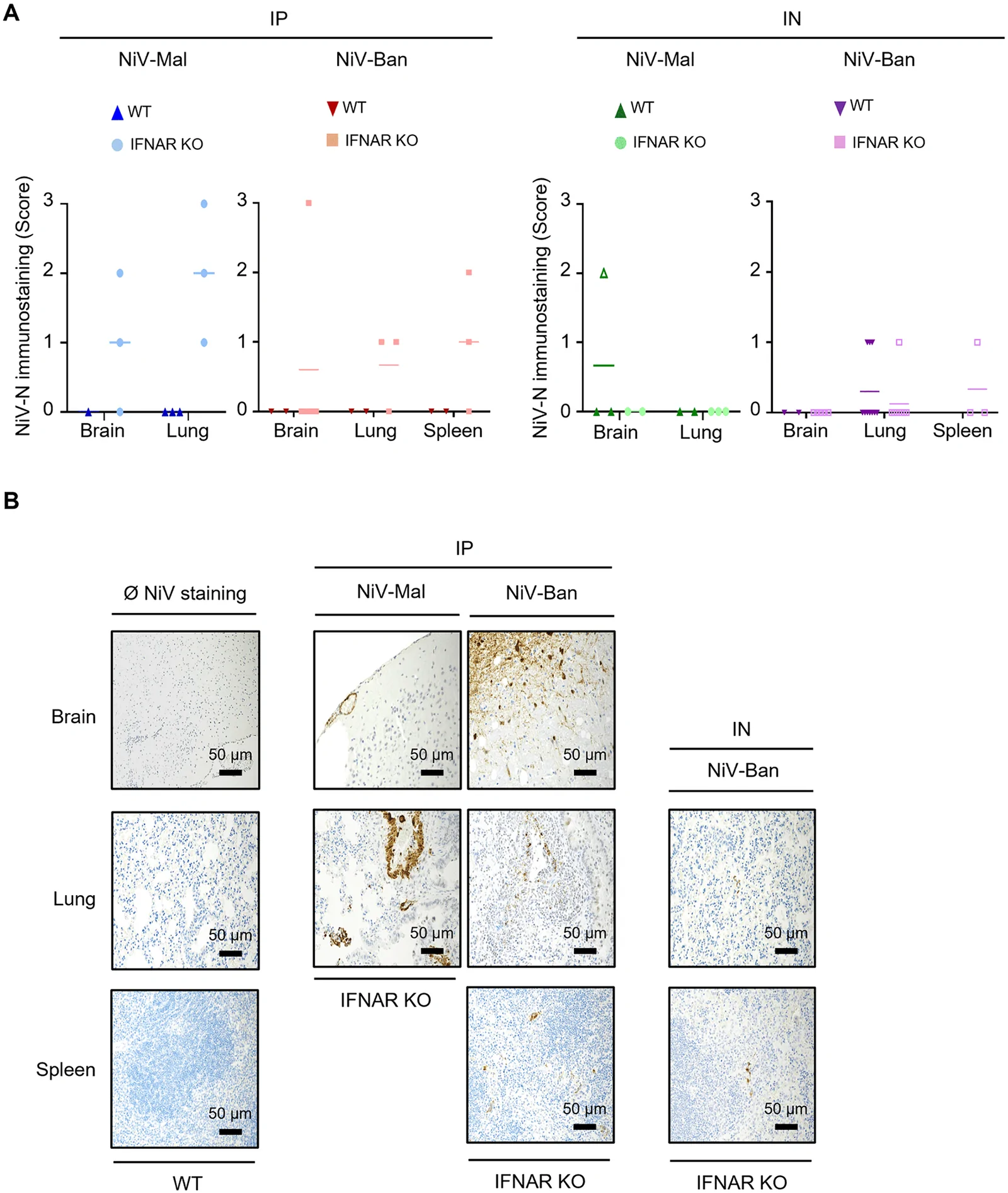
NiV spread tends to be higher following intraperitoneal inoculation compared to intranasal inoculation in IFNAR KO mice. Organs harvested from animals positive for NiV-N RNA were included in paraffin and sections were stained with a rabbit polyclonal anti-NiV-N antibody and DAPI for immunohistochemistry analysis. **(A)** A scoring system was applied by a veterinary pathologist (0 = no labelling; 1 = a few positive cells; 2 = small clusters of positive cells; 3 = multiple large or extensive foci of positive cells) on slides obtained from IP- (left panel) or IN-inoculated (right panel) organs, respectively. Empty dots represent animals that succumbed or were euthanized during early, mid or late phases of infection (until day 10 for the late phase). **(B)** Brain, lung and spleen sections from WT mice were used as NiV-negative control conditions while similar sections from IFNAR KO mice were used to characterize the presence of NiV-N antigen following infection. Scale bar represents the size of analyzed portions.

**Fig 6:**
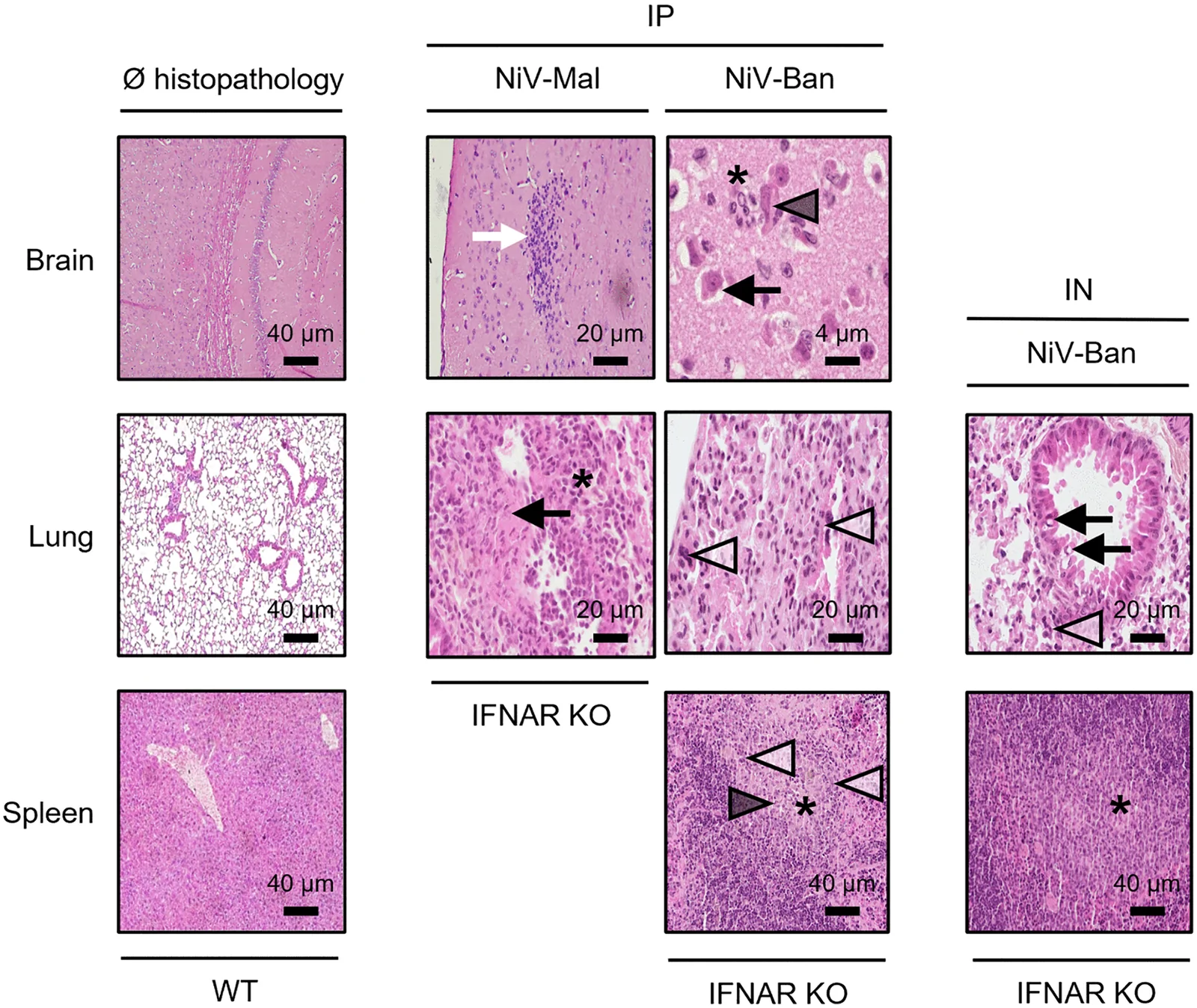
Intraperitoneal inoculation may generate extended immunopathogenesis compared to intranasal inoculation in IFNAR KO mice challenged by NiV. Organs harvested from animals positive for NiV-N RNA underwent hematoxylin-eosin-saffron (HES) staining and were evaluated by a board-certified veterinary pathologist. Brain, lung and spleen sections from WT mice were used as control conditions while similar sections from IFNAR KO mice were used to characterize the presence of immunopathogenic manifestations following NiV infection. White arrow: granulocyte infiltration; Black arrow: inclusion bodies; Black star: hystiocytosis; White arrowhead: syncytial cells; Black arrowhead: necrotic inclusion bodies. Scale bar represents the size of analyzed portions.

To investigate NiV-Mal- and NiV-Ban-related immunopathogenesis, we performed a histopathological analysis and immunolabeling of NiV-N antigen on brain, lung and/or spleen sections from selected animals that displayed pathological signs and succumbed or were euthanized (Figs 5, 6, S3A and S3B). In parallel, NiV-Mal IP-inoculated WT mice, that all survived the challenge and cleared the virus by d32, were used as control conditions (Figs 5, 6, S3A and S3B). Indeed, no viral antigen was detected in any section from WT animals and no major histological signs were displayed (Figs 5A, left panel and S3A).

Regarding the samples from NiV-Mal-IP-inoculated IFNAR KO mice, all were positive for NiV-N labeling (Fig 5A, left panel and 5B). Specifically, viral antigens detected in the brain were localized in meningeal vessels and the olfactory bulb, respectively, associated with a characterized infiltration of granulocytes (Fig 5A, left panel and 5B). For the selected lung sections, NiV-N antigens were localized in bronchial epithelium, notably in pneumocytes (Fig 5B). Interestingly, when investigating inflammatory pathogenic signs, animals that were positive for NiV-N labeling displayed characteristic vascular lesions including syncytial cells in the endothelium, vascular wall damage, perivascular inflammation and edema (Fig 6).

Concerning NiV-Ban-IP-inoculated IFNAR KO mice, three out of four animals were positive to NiV-N antigen (Fig 5A, left panel) with a cellular tropism associated to pneumocytes in the lungs and large mononuclear cells of the marginal zone in the spleen (Fig 5B) in only two of these positive animals. In parallel, immunopathogenic evaluations revealed that NiV-positive lung tissues were associated with perivascular inflammation and diffused syncytial cells throughout alveoli, vessels and bronchi (Fig 6). Regarding the spleen, syncytia and necrosis were characterized mainly in expanded marginal zones rich in mononuclear cells, thus displaying histiocytosis (Fig 6).

Regarding IN-inoculated animals, as viral RNA was detected in few samples and almost exclusively from NiV-Ban infected IFNAR KO mice outside the lung sections at the time of final euthanasia, we only selected positive animals for further immunohistochemistry analysis (Figs 5A, right panel, 5B, 6, S3A and S3B). Indeed, 5 out of 20 mice with detectable viral RNA were positive for NiV antigen labeling, thus confirming that RNA detection is more sensitive than protein labeling (Figs 5A, right panel and 5B). Importantly, our investigations led to similar conclusions that independently of the murine genotype, the major histological observation was the presence of perivascular inflammatory cell infiltration in the lungs and focal expansion of the marginal zone in the spleen (Figs 6 and S3B). Interestingly, the WT mouse that succumbed to IN-inoculated NiV-Mal at d20 was positive for NiV antigen in the brain and displayed focal encephalitis associated with histiocytosis (S3B Fig). Neither viral antigen nor histopathological lesions were detected in the brain of any other IN-inoculated animal. In general, we observed that independently of the viral strain, IP inoculation led to greater pathogenesis than IN inoculation. Then, our preliminary investigation showed that NiV-Ban IP-inoculated IFNAR KO mice could present virus-induced immunopathology, although to a lesser extent compared to NiV-Mal. Also, IN inoculation tended to perpetrate more severe immunopathogenesis following NiV-Ban infection in comparison to NiV-Mal. Overall, this exploratory work describes IFNAR KO mice as an affordable small animal model with many genetic tools available, to study both NiV-Mal and NiV-Ban infection.

## Discussion

More than 25 years after its first isolation, NiV remains a global threat due to its high lethality, its human-to-human transmission and the lack of approved therapeutics. Both NiV-Mal and NiV-Ban strains are associated with different symptoms and pathogenicity [39]. It is possible that the CFR associated to outbreaks is influenced by multiple factors, such as the viral dose of infection and the route of transmission, as well as socio-economic factors such as Public Health measures and access to healthcare [8,40,41]. Indeed, while NiV-Mal emerged only twice in 1998–1999 and 2014, NiV-Ban re-emerges frequently since 2001 [42]. Nonetheless, two bats in a city market in Central Java, Indonesia, have been reported positive to NiV-Mal recently, thus highlighting the risk of spillover from both viral strains [43]. The causes responsible for the different diseases associated to NiV-Mal- and NiV-Ban-infected patients remain unclear, and multiple animal models have been tested to characterize its respective pathogenesis [25,28,29,44–51]. While non-human primates, hamsters and ferrets succumb to NiV infection, WT mice clear the virus despite being permissive [25]. However, we previously demonstrated that IFNAR KO mice succumb to NiV-Mal intraperitoneal challenge due to the lack of type I IFN signaling [25]. So far, experiments involving NiV-Ban infection in murine models are of interest to evaluate pathogenic differences between both viral strains *in vivo*. Here, we investigated potential differences between NiV-Mal and NiV-Ban infection in both WT and IFNAR KO mice following IP and IN routes of virus inoculation.

The exploratory nature of this work opens a window regarding the implementation of a robust small animal model to study NiV-related pathogenesis. However, there are several limitations in the presented study and some results represent preliminary tendencies that will require further experiments to be validated (Figs 3-6 and S3). Indeed, to reach significant number of mice in the kinetic studies, animals analyzed at different individual time-points were pooled in three groups defined as “early”, “mid” and “late” phases (Figs 3 and 4) representing the pre-critical (incubation), critical (pathogenic) and post-critical (control/recovery) periods of infection, respectively, as previously defined in NiV-infected IFNAR KO mice [27]. Moreover, as heterogeneity in the outcomes involving surviving and euthanasia of animals occurred in some of these groups, cautious interpretations were made regarding survival and organ-associated pathogenesis. Importantly, our work led to similar observations made and results obtained concomitantly [30].

First, we demonstrated that WT and IFNAR KO primary MEFs are permissive to both NiV-Mal and NiV-Ban infections. Our experiments determined notable differences in the kinetic and the size of formed syncytia with IFNAR KO cells being more prone to viral dissemination compared to WT cells. Also, our initial *in vitro* tests described that NiV-Mal expansion is more efficient than NiV-Ban, thus confirming previous observations made in a hamster model [44,46]. Then, we investigated such disparities *in vivo* by challenging WT and IFNAR KO mice with either NiV strain through IP or IN route of inoculation. Indeed, consistent with *in vitro* and *in vivo* observations [30], higher lethality was observed in IFNAR KO animals following IP-inoculated-NiV-Mal compared to IP-inoculated-NiV-Ban, while all WT mice survived. Surprisingly, all IFNAR KO mice including animals infected with NiV-Ban, known to be associated with respiratory distress syndrome in humans, survived IN inoculation. Indeed, contrary to IP route, IN inoculation led to a subclinical infection with either NiV strain in IFNAR KO mice, thus confirming several observations where the IN route lead to an attenuated disease [25,30]. Noteworthy, the lower inoculation dose (10^5^ PFU) used for IN challenge in this study compared to the dose used in our previous report (10^6^ PFU) might be responsible for the absence of fatal outcomes [25], thus relevant with the use of 10^6^ TCID_50_ that led to a higher mortality [30]. Overall, contrary to the human-associated NiV disease observed in African green monkeys, rodents including our murine models may fail to recapitulate NiV morbidities and outcomes [44,50], potentially due to species-specific factors, such as a more developed basal innate immune environment in the respiratory tract compared to primates [52–54].

Concomitantly, IN-inoculated and surviving IP-inoculated IFNAR KO mice produced significantly higher nAb titers compared to WT mice following NiV infection with either strain. This shows that despite the lack of IFN-I signaling-dependent activity and higher viral replication, the adaptive immune response might be strong enough to control viral growth. Interestingly, the lack of extensive visible pathogenic signs in both WT and IFNAR KO mice following IN inoculation may be related to a major role of type III IFNs in the defense against pathogens in mucosal barriers such as the respiratory tract, as previously described [55–57]. Regarding brain infection, few analyzed samples were positive following IN inoculation, suggesting potential differences in viral entry in the central nervous system (CNS) through the olfactory bulb as described in hamsters [58]. Intriguingly, in the mouse model, the hematogenous route resulted in a higher propensity of the virus to reach the CNS, as observed in IP-inoculated mice. Globally, considering the absence of clinical signs of disease and scarce viral propagation, murine models do not appear to be very susceptible to IN inoculation of NiV. However, further tests are needed with a higher dose or with combined KOs clarify differences between NiV-Mal and NiV-Ban *in vivo* following IN inoculation.

Interestingly, a difference in cell tropism was observed in the lungs between the two viral strains following IP inoculation in our work. Indeed, NiV-Mal-associated antigen was mainly detected in the bronchi while NiV-Ban was associated with alveolar pneumocytes. Moreover, syncytia formed in the lungs were centered on blood vessels with NiV-Mal whereas they were diffused throughout lung parenchyma and bronchi with NiV-Ban. However, additional investigations are necessary to understand whether the observed cellular tropism and syncytia distribution are host-specific and whether they exert a role on viral shedding. Furthermore, virus-induced cell-to-cell fusion has been demonstrated to be associated with STING responses and might contribute to disrupt the blood-brain barrier following NiV infection as well, but syncytia formation is not always analyzed in animal experiments in the literature [27,59–61].

Concerning the spleen, our observations described that two IFNAR KO animals that succumbed to NiV-Ban IP inoculation displayed foci of NiV-N antigen-positive macrophages as previously observed in different tissues [11,46,62]. Further investigations aimed at characterizing the precise role(s) of macrophages during NiV infection are of high interest to understand its involvement as targets for viral replication or key immune partners to control infection.

In the brain, we did not notice strain-specific differences in cell tropism and viruses were found in diverse compartments. This corroborates the observation that NiV can potentially distribute and induce lesions throughout the whole brain, as recently reported by Goldin *et al*. following NiV-Ban infection in AGMs [63]. Moreover, while both viral strains could induce similar lesions, the capacity of NiV-Ban to reach the brain in humans might be restrained due to the induction of fast and severe respiratory syndrome, thus limiting its ability to further affect the brain. Indeed, this hypothesis was supported by Goldin *et al.*, who reported that treatments counteracting NiV-Ban respiratory disease ultimately led to the death of monkeys through encephalitis [63].

Altogether, we evaluated IFNAR KO mice as a potential experimental model for both NiV-Mal and NiV-Ban infections. IP inoculation resulted in lethal outcome in 100% of NiV-Mal- and 60% of NiV-Ban-infected mice in this model with viral replication detected in all tested organs. Conversely, IN inoculation induced a subclinical infection with either viral strain. While hamsters and AGMs are the most suitable models to study NiV infection due to their natural susceptibility to the virus, mice could represent a useful alternative due to the large number of immune reagents, genetic tools and global knowledge in murine immune system available. The characterization of a possible animal model to study long-term infection is of high importance and further studies on NiV-Ban-infected IFNAR KO mice inoculated through IP could be interesting as high levels of viral RNA were detected at late time points despite the absence of signs of disease. Moreover, the possibility to produce specific KO mice may allow further dissection of specific immune factors involved in the control of NiV. In conclusion, our exploratory analysis paves the way in evaluating mice as a potentially valid small animal model for specific immunopathogenic and fundamental investigations in the study of NiV infection.

## Supporting information

S1 Fig Experimental design of in vivo studies. (A) 5 WT mice and 6 IFNAR KO mice were intraperitoneally (IP) inoculated with 10^6^ PFU of NiV-Mal and monitored during 32 days. (B) 5 WT and 5 IFNAR KO mice were intranasally (IN) infected with 10^5^ PFU of NiV-Mal and monitored during 32 days. (C) 5 WT and 5 IFNAR KO mice were IP-infected with 10^6^ PFU of NiV-Ban and monitored during 32 days. Moreover, 20 WT and 12 IFNAR KO mice IP-infected with 10^6^ PFU of NiV-Ban were sacrificed at programmed time points: day 0 + 4 hours, 1, 2, 4, 7 and 10 (2 or 3 animals per time point). (D) 5 WT and 5 IFNAR KO mice were IN-infected with 10^5^ PFU of NiV-Ban and monitored during 32 days. Moreover, 20 WT and 15 IFNAR KO mice IN-infected with 10^5^ PFU of NiV-Ban were sacrificed at programmed time points: d0 + 4h, 1, 2, 4, 7 and 10 (2 or 3 animals per time point). (E) Brains, lungs, spleens and livers from all animals were harvested at the day of death, programmed euthanasia or experimental endpoint and were processed though RNA extraction and real time RT-qPCR. (F) Brains, lungs and spleens from selected animals underwent hematoxylin and eosin staining (HES) and immunohistochemistry with α-NiV-N antibody. Created in BioRender. CIRI, I. (2026) https://BioRender.com/i4l85rv.
(TIF)

S2 Fig Different pathogenic signatures of NiV Mal and NiV-Ban infection in mice *in vivo.* WT and IFNAR KO mice were infected through an intraperitoneal route (A, C) or intranasal route (B, D) with 10^6^ or 10^5^ PFU, respectively, of NiV-Mal or NiV-Ban. All groups were constituted of 5 animals except for NiV-Mal IFNAR KO group, which consisted of 6 mice. Animals were monitored for 32 days for weight (A-B) and clinical signs with established clinical scores (C-D).
(TIF)

S3 Fig Intraperitoneal and intranasal NiV infections are attenuated and generate mild immunopathogenesis in WT mice. (A) Organs from selected IN-infected animals were included in paraffin and sections were stained with a rabbit polyclonal anti-NiV-N antibody and DAPI for immunohistochemistry analysis. (B) Organs from selected animals underwent hematoxylin-eosin-saffron (HES) staining and were evaluated by a board-certified veterinary pathologist. Brain and lung and sections from WT mice were used as NiV-negative control conditions while similar sections from WT mice sensitive to NiV infection were used to characterize the presence of NiV-N antigens (A) or immunopathogenic manifestations (B). Black star: hystiocytosis; White arrowhead: syncytial cells. Scale bar represents the size of analyzed portions.
(TIF)

S1 Data Raw data. File containing the raw data presenting all numerical or measurable parameters from all figures. The values associated to the growth kinetics of NiV-Mal and NiV-Ban are presented (Fig 1 sheet, panels E and F). Numerical values of the survival curves and sero-neutralization assays are listed (Fig 2 sheet). Raw amounts of NiV-N RNA levels in the brain, lungs, spleen and liver from IP- and IN-inoculated mice are shown (in both Fig 3 and 4 sheets, respectively). Numerical scores of NiV-N protein staining from brain, lung and spleen sections defined by pathologists are presented (Fig 5 sheet, panel A). Evaluations and comments from each selected section by pathologists are listed (Fig 5 sheet, panel B, Fig 6 and S3 sheets). Weights and clinical scores associated to each individual animal are presented (S2 Fig sheet).
(XLSX)
